# Synthesis and X-ray Structural Studies of a Substituted 2,3,4,5-Tetrahydro-1*H*-3-benzazonine and a 1,2,3,5-Tetrahydro-4,3-benzoxazonine

**DOI:** 10.3390/molecules20010487

**Published:** 2014-12-31

**Authors:** Timothy S. Bailey, John B. Bremner, Brian W. Skelton, Allan H. White

**Affiliations:** 1 Department of Chemistry, University of Tasmania, Hobart, Tas. 7001, Australia; 2 Tasmanian Alkaloids Pty Ltd, P.O. Box 130, Westbury, Tas. 7303, Australia; E-Mail: SBAILEY3@its.jnj.com; 3 School of Chemistry, University of Wollongong, Wollongong, NSW 2522, Australia; 4 School of Chemistry and Biochemistry M310, The University of Western Australia, Crawley, WA 6009, Australia; E-Mails: brian.skelton@uwa.edu.au (B.W.S.); allan.white@uwa.edu.au (A.H.W.); 5 Centre for Microscopy, Characterisation and Analysis, M010, The University of Western Australia, Crawley, WA 6009, Australia

**Keywords:** large rings, X-ray crystal structures, benzazonine, benzoxazonine

## Abstract

Using a common 1-(1-phenylethenyl)-1,2,3,4-tetrahydroisoquinoline precursor to the required ylide or *N*-oxide intermediate, the Stevens [2,3] and analogous Meisenheimer [2,3] sigmatropic rearrangements have been applied to afford concise syntheses of phenyl -substituted representatives of each of the reduced 1*H*-3-benzazonine and 4,3-benzoxazonine systems, respectively. Single crystal X-ray structure determinations were employed to define the conformational characteristics for each ring type.

## 1. Introduction

Annulated medium-ring heterocycles have attracted significant research attention owing to the vast array of systems possible with potential for different chemical and biological properties [1,2,3,4]. For example, some 3-benzazonines with a nine-membered ring show 5-HT_2A_ antagonist activity [5], while the eight-membered ring-containing benzoxazocines display a range of biological properties including analgesic [6] and NK_1_ (neurokinin receptor) inhibitory activity [7]. Synthetic approaches to these systems often involve rearrangement strategies incorporating a ring expansion [8,9,10,11], although ring formation [12,13,14] and ring cleavage approaches [5,15,16] can also be used. The Meisenheimer [1,2] and [2,3] sigmatropic rearrangements of amine *N*-oxides [17,18,19] and the analogous Stevens [2,3] sigmatropic rearrangements of ylides [20,21] afford good opportunities for medium ring synthesis. To further explore these synthetic prospects and to assess substituent effects (e.g., a phenyl group *vs.* a methyl group or hydrogen [17]) on configurational and conformational issues in the products, we have investigated the synthetic utility of the [2,3] versions of these rearrangements from the same starting amine precursor, the 1-(1-phenylethenyl) substituted tetrahydroisoquinoline derivative **3**. These rearrangements involve three-atom ring expansions with incorporation of an O-C-C (Meisenheimer) or C-C-C (Stevens) unit into the six-membered N-containing ring. Soldatenkov* et al.* have also reported that a tetrahydroisoquinoline *N*-ylide can undergo a somewhat related [1,4] sigmatropic rearrangement on reaction with dimethyl acetylenedicarboxylate affording a 3-benzazonine derivative in good yield [22]. The results of our work and the single crystal X-ray structural analyses of the benzo-fused nine-membered ring heterocycles produced are discussed in this paper.

## 2. Results and Discussion

### 2.1. Synthesis

**Scheme 1:**
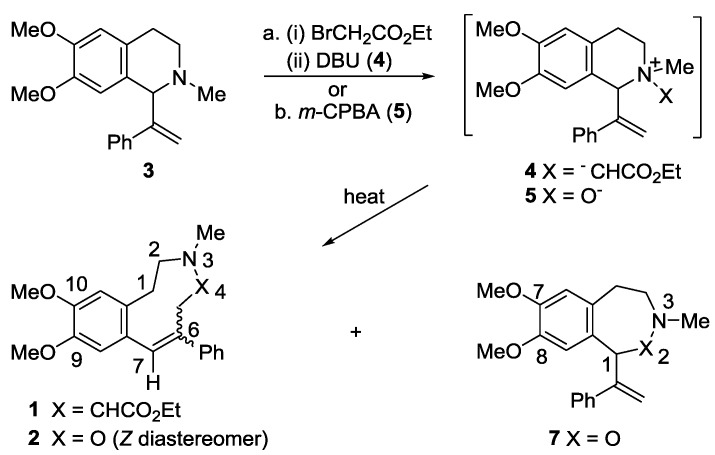
Synthesis of the benzazonine **1** and benzoxazonine **2**.

The synthesis of the fused medium ring compounds **1** and **2** proceeded from the common starting tetrahydroisoquinoline derivative **3** (Scheme 1), readily accessible in turn by nucleophilic addition of the Grignard reagent 1-phenylethenylmagnesium bromide to the known salt 6,7-dimethoxy-2-methyl-3,4-dihydroisoquinolinium iodide [23].

**Figure 1:**
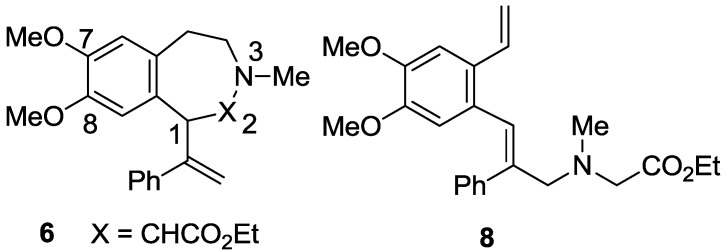
Proposed structures for the benzazepine **6** and ring-opened compound **8**.

Reaction of the tetrahydroisoquinoline **3** with ethyl bromoacetate gave a diastereomeric mixture of the quaternary salts which, on treatment with DBU in acetonitrile at 25 °C, gave mainly the nine-membered 3-benzazonine **1** as a mixture of the *E* and *Z* diastereomers from the ylide **4** and a Stevens [2,3] rearrangement. A very minor amount of the Stevens [1,2] rearrangement product **6** (Figure 1, 8% yield based on ^1^H-NMR analysis) was also formed as an inseparable mixture (HPLC) with a trace of another compound tentatively assigned as the isomeric ring-opened product **8** (Figure 1); a little more of both **6** and **8** were formed on repeating the reaction in acetonitrile heated at reflux but they could not be separated. The structure of the benzazepine **6** followed from the ^1^H-NMR spectral data on the mixture, particularly the characteristic coupled doublet signals ascribed to H1 (3.66 ppm) and H2 (4.50 ppm), while for the ring-opened product **8**, diagnostic signals consistent with an isolated mono-substituted vinyl group, a separate olefinic proton, and two isolated N-methylene protons (singlets at 3.61 and 3.15 ppm in the ^1^H-NMR and at 58.3 and 55.2 ppm in the ^13^C-NMR) were apparent. Potentially this latter product could arise via a thermal 1,3-rearrangement [24,25] of the tetrahydroisoquinoline **3** to an eight-membered 5‑phenyl-3-benzazocine intermediate and subsequent N-quaternization followed by a DBU-mediated Hofmann elimination to give **8** with the *E* geometry about the stilbenic double bond.

The diastereomers of the benzazonine **1** were separated by preparative HPLC and their structures were elucidated initially on the basis of NMR, mass spectrometric and elemental analytical data. Consistent with the nine-membered ring structure was the presence of a singlet signal downfield at 6.91 ppm in the ^1^H-NMR (C_6_D_6_ room temperature; ^13^C-NMR, C7 at 130.5 ppm) or 6.74 ppm (C_6_D_6_, 75 °C; ^13^C-NMR, C7 at 131.5 ppm) ascribed to the vinylic proton H7 in the *E* and *Z* diastereomers, respectively. The *E* *vs. Z* stereochemistry was assigned on the basis of a nOe interaction between H7 and the *ortho*-6-phenyl protons in the *E*-**1** diastereomer but not the *Z*-**1** diastereomer. The benzazonine *Z*-**1** exhibited conformational flexibility on the basis of the ^1^H-NMR spectral analysis at 25 °C in either C_6_D_6_ or CDCl_3_. Broad peaks lacking definition were observed. At 75 °C in C_6_D_6_ a single set of signals was obtained for *Z*-**1** which supported the proposed structure. At this temperature only the signals ascribed to H5 and the ethyl ester protons displayed poor peak shape. At −50 °C in CDCl_3_, two conformations in a ratio of about 1.94:1 appeared, with substantially different diagnostic singlet signals at 2.36 and 2.69 ppm (N-Me group) and multiplet signals at 1.41–1.35 and 0.87–0.81 ppm (ester methyl group) for the major and minor conformations, respectively.

Conversion of the tetrahydroisoquinoline **3** to the *N*-oxide derivative was achieved by oxidation with *m*-CPBA in dichloromethane at room temperature to afford the *trans*-*N*-oxide **5** (53%), together with a substantial yield of a mixture (up to 25%; 44:56 ratio) of the isomeric rearranged products **2** and **7**. Only the *Z*-diastereomer of the nine-membered ring benzoxazonine **2** was observed. Similar results were obtained on oxidation with cooling of the solution during the reaction and on work up. The Meisenheimer [2,3] rearrangement product **2** and the Meisenheimer [1,2] rearrangement product **7** were isolated by preparative HPLC. In the ^1^H-NMR of the benzoxazonine **2** at 25 °C in CDCl_3_, the H7 olefinic proton appeared as a downfield singlet at 7.00 ppm, while the H1 and H2 methylene proton signals were broad and integrating as only 2–3 protons. At low temperature (−30 °C), two conformers of the benzoxazonine **2** (*ca.* 3.5:1 ratio) were apparent in the ^1^H and ^13^C-NMR spectra, which were probably related by the inversion or “flipping” of the N3-O4-C5 segment of the medium ring. With the seven-membered ring product **7**, downfield singlet signals at 5.98 and 5.53 ppm in the ^1^H-NMR were consistent with the presence of the methylidene group, while H1 was ascribed to the singlet signal further upfield at 5.15 ppm.

Furthermore, a solution of the benzoxazonine **2** in acetonitrile heated at reflux led to slow isomerisation via a [1,3] shift to yield the benzoxazepine **7**; an equilibrium between **2** and **7** (*ca.* 24:76 ratio, **2**:**7**) was obtained on heating either** 2** or **7** at a higher temperature in refluxing xylene for 1 h.

In both the Stevens and Meisenheimer [2,3] rearrangements, the *cis* isomer of the respective intermediate *N*-ylide **4** or *N*-oxide **5** is involved, as a favourable concerted transition state geometry [19,21] can be accessed to give the nine-membered ring products. On the other hand, with the corresponding *trans*-isomeric ylide or *N*-oxide, this is not the case, and the diradical mediated [1,2] rearrangements to give seven-membered ring products can then proceed. Interestingly, with a 1-ethenyl or 1-isopropenyl substituent in the tetrahydroisoquinoline precursor of the corresponding ylide, only the Stevens [2,3] rearrangement products were observed, while the analogous *N*-oxides gave only the Meisenheimer [1,2] rearrangement product (from the 1-ethenyl precursor) or a mixture of the [1,2] and [2,3] rearrangement products (from the 1-isopropenyl precursor) on heating at reflux in acetonitrile for 50 min [17].

### 2.2. X-ray Structural Studies

To support the structural elucidations, single crystals of the 3-benzazonine *E*-**1** and of the 4,3-benzoxazonine **2** were obtained for X-ray crystallographic analysis. Suitable crystals for an X-ray study of *Z*-**1** could not be obtained.

**Figure 2:**
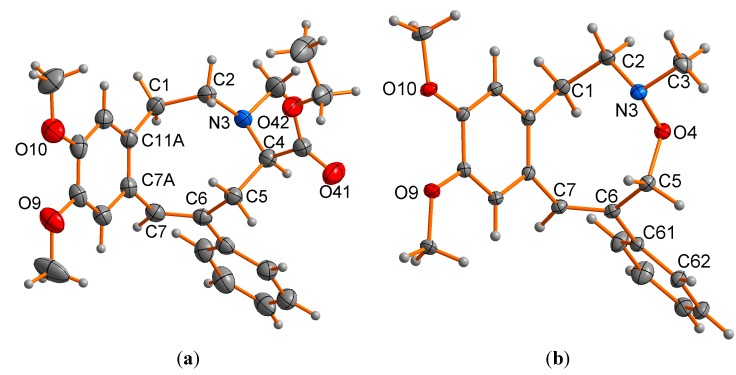
Molecular projections of *E*-**1** (**a**); **2** (**b**), normal to the “planes” of their “macrocycle” rings; the *R* enantiomer, arbitrarily adopted as the asymmetric unit in a centrosymmetric space group, is shown in (a).

The results of the single crystal X-ray studies on *E*-**1** and **2** were consistent with the above formulations in terms of stoichiometry and connectivity (Figure 2a,b, Table 1, Table 2 and Table 3), establishing solid-state conformations, and with bond distances and angles generally conforming to expected norms. Counterpart values and figures for two closely related systems (CCDC: AYEKOF [26], SELXUC [27]; see Figure 4 for structures) are also included for comparison.

**Figure 3:**
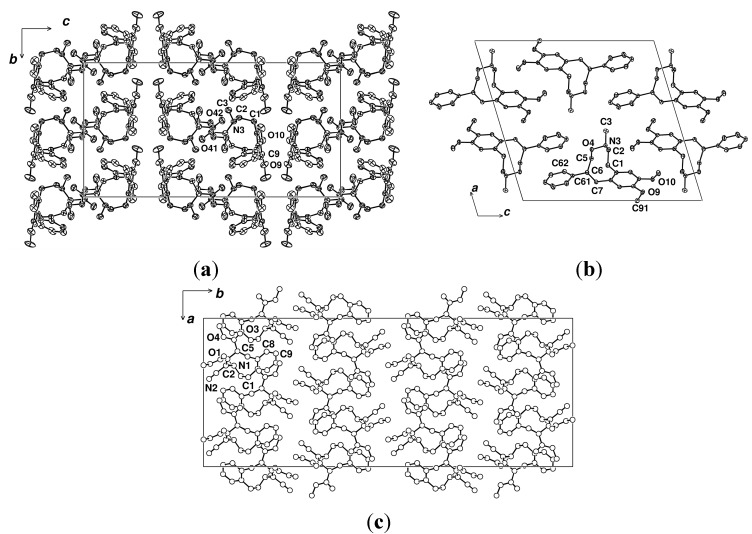
(**a**) Unit cell contents of *E*-**1**, projected down *a*, showing the layering normal to* c*; (**b**) Unit cell contents of **2**, projected down *b*; (**c**) Unit cell contents of AYEKOF [26], projected down *c*, showing the layering normal to *b* (*cf.* (a)).

**Figure 4:**
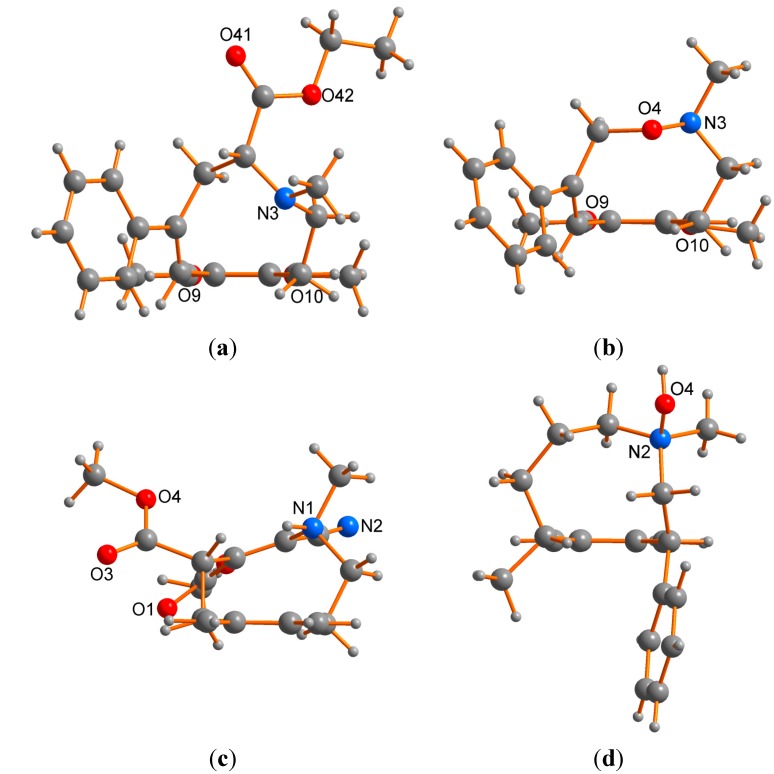
The molecular cores of *E*-**1** (**a**); **2** (**b**); AYEKOF [26] (**c**) and SELXUC [27] (**d**); projected through the parent aromatic ring as a “baseline” at the bottom of the “macrocycle” of each picture, and displaying the separate macrocycle conformations.

Notes: In AYEKOF, the protonating hydrogen atom at the nitrogen atom, although shown in Figure 1 of reference [26], is not included in the CCDC cif file, and was restored as an estimate by us for the purpose of the present Figure; the macrocycle double bond is located on the other side of the ring nitrogen atom, between it and the parent phenyl ring in this species.

The crystal packings are of some interest, with that of *E*-**1** comprising sheets normal to *c* (Figure 3a), similar to that normal to *b* observed with AYEKOF [26] (Figure 3c), while **2** is a simple translational stacking up *b* (Figure 3b). The fused aromatic rings with their *ortho-*methoxy substituents are unremarkable, the methyl groups lying *quasi*-coplanar with the aromatic ring with the usual exocyclic angle asymmetries at the pendant bonds, and their phenyl pendants *quasi*-normal to the supporting phenyl ring plane (Figure 4a,b).

The principal interest in the two structures *E*-**1** and **2** lies in the novelty of their macrocycle components. There are diverse analogues of *E*-**1** structurally characterized in the literature, perhaps the most relevant benzo-fused systems with similarly located nitrogen atoms being those of a 1α,3α,7β-2,3,4,5,6,7-tetrahydro-3,7-dimethyl-1-phenyl-1*H*-3-benzazonine *N*-oxide-saccharin complex [27] [(CCDC: SELXUC)], albeit with a more heavily substituted nitrogen atom and lacking the double bond, and of dimethyl 4-cyano-3-methyl-2,3,6,7-tetrahydro-1*H*-3-benzazonine-5,6-dicarboxylate [26] [(CCDC: AYEKOF)], also with a quaternary nitrogen atom with the C=C bond adjacent to it (rather than distant) in the ring. To the best of our knowledge, there are no other X-ray crystallographic studies on derivatives with the 4,3-benzoxazonine system as in **2**.

**Table 1:** “Macrocycle” torsion angles (degrees) (*E***-1**, **2**; counterpart values for (CCDC) AYEKOF [26], SELXUC [27] are also given.). Atoms are denoted by number only, *N italicized*; note that atom 4 in **2** is oxygen.

Compound	*E*-1	2	AYEKOF [26]	SELXUC [27]
11a-1-2-*3*	105.2 (2)	52.56 (12)	−68.4 (2)	−43.3 (3)
1-2-*3*-4	−105.0 (2)	63.90 (11)	−64.1 (2)	75.3 (3)
2-*3*-4-5	55.8 (2)	−122.48 (8)	104.4 (2)	50.8 (2)
*3*-4-5-6	59.0 (2)	113.25 (9)	2.8 (3)	−109.4 (2)
4-5-6-7	−118.1 (2)	−90.31 (12)	−96.1 (2)	54.2 (3)
5-6-7-7a	0.9 (2)	4.0 (2)	110.4 (2)	−72.1 (3)
6-7-7a-11a	70.0 (2)	69.59 (14)	−76.7 (2)	105.4 (3)
7-7a-11a-1	2.1 (2)	−3.32 (15)	−2.4 (3)	4.3 (3)
7a-11a-1-2	−91.6 (2)	−109.39 (11)	104.4 (2)	−60.4 (3)

**Table 2:** Selected geometries (*E*-**1**, **2**; counterpart values for AYEKOF (100 K) [26], SELXUC (293 K) [27] are also given.) *.

Atoms	Parameter (Macrocycle)	Atoms	Parameter (Phenyl Ring)
Distances (Å)			
C(1)-C(2)	1.544(2), 1.5259(15), 1.522(3), 1.539(3)	C(7a)-C(8)	1.409(2), 1.4105(14), 1.401(3), 1.389(4)
C(1)-C(11a)	1.512(2), 1.5129(14), 1.513(3), 1.521(2)	C(7a)-C(11a)	1.389(2), 1.3918(13), 1.405(3), 1.406(4)
C(2)-N(3)	1.475(2), 1.4588(14), 1.483(3), 1.504(3)	C(8)-C(9)	1.370(2), 1.3834(13), 1.389(3), 1.357(4)
N(3)-C,*O*(4)	1.450(2),*1.4544(10)*, 1.431(3), 1.518(4)	C(9)-C(10)	1.396(2), 1.4101(13), 1.383(3), 1.370(5)
N(3)-C(3)	1.459(2), 1.4590(14), 1.470(3), 1.491(3)	C(10)-C(11)	1.380(2), 1.3836(13), 1.388(3), 1.381(4)
C,*O*(4)-C(5)	1.533(2),*1.4453(12)*, 1.346(3), 1.517(4)	C(11)-C(11a)	1.407(2), 1.4094(13), 1.397(3), 1.390(3)
C(5)-C(6)	1.509(2), 1.5106(13), 1.505(3), 1.522(4)	C(9)-O(9)	1.371(2), 1.3683(11), -, -
C(6)-C(7)	1.340(2), 1.3431(13), 1.555(3), 1.534(4)	C(10)-O(10)	1.370(2), 1.3651(11), -, -
C(7)-C(7a)	1.483(2), 1.4876(13), 1.511(3), 1.515(3)		
C(6)-C(61)	1.488(2), 1.4892(13), 1.524(3), -		
Angles (degrees)			
C(11a)-C(1)-C(2)	115.71(13), 113.11(8), 113.9(2), 117.2(2)	C(7)-C(7a)-C(8)	117.16(14), 118.12(8), 118.5(2), 118.6(3)
C(1)-C(2)-N(3)	113.79(13), 111.51(8), 113.5(2), 116.3(2)	C(7)-C(7a)-C(11a)	123.75(14), 122.46(9), 122.7(2), 123.4(2)
C(2)-N(3)-C,*O*(4)	117.61(13), 106.90(7), 112.0(2), 115.8(2)	C(8)-C(7a)-C(11a)	119.09(14), 119.29(8), 118.8(2), 117.9(2)
C(2)-N(3)-C(3)	113.73(13), 111.35(9), 110.0(2), 111.6(2)	C(7a)-C(8)-C(9)	121.82(16). 121.48(9), 121.4(2), 122.6(3)
C,*O*(4)-N(3)-C(3)	112.03(12), 105.62(8), 112.3(2), 111.2(2)	C(8)-C(9)-O(9)	125.26(16), 125.56(9), -, -
N(3)-C,*O*(4)-C(5)	112.08(12), 108.87(7), 121.5(2), 113.7(2)	C(10)-C(9)-O(9)	115.41(15), 115.25(8), -, -
C,*O*(4)-C(5)-C(6)	113.93(13), 112.02(8), 120.9(2), 118.1(2)	C(8)-C(9)-C(10)	119.32(15), 119.19(9), 119.7(2), 119.6(3)
C(5)-C(6)-C(7)	121.28(14), 121.43(9), 113.3(2), 118.3(3)	C(9)-C(10)-C(11)	119.51(15), 119.44(9), 119.5(2), 119.9(2)
C(5)-C(6)-C(61)	118.25(13), 118.33(8), 111.3(2), -	C(9)-C(10)-O(10)	114.75(15), 115.29(8), -, -
C(61)-C(6)-C(7)	120.31(14), 120.24(9), 112.3(2), -	C(11)-C(10)-O(10)	125.73(16), 125.27(9), -, -
C(6)-C(7)-C(7a)	124.86(14), 126.88(9), 112.44(14), 110.8(2)	C(10)-C(11)-C(11a)	121.66(16), 121.54(9), 121.6(2), 121.1(3)
		C(11)-C(11a)-C(7a)	118.57(15), 119.00(9), 119.0(2), 118.9(2)
		C(11)-C(11a)-C(1)	118.73(14), 118.00(8), 118.0(2), 117.6(2)
		C(7a)-C(11a)-C(1)	122.70(14), 122.98(8), 123.0(2), 123.5(2)
		C(9)-O(9)-C(91)	115.93(14), 116.82(8), -, -
		C(10)-O(10)-C(101)	117.91(15), 116.86(8), -, -

* For the oxygen atom pendant at N(3), N-O is 1.4275(14) Å, O-N-C(9,10,3) are 102.19(12), 107.16(13), 108.11(15)°. For the methyl group pendant at C(7), (H_3_)C-C(7) is 1.529(4) Å; C-C-C(6,7a) are 110.7(3), 111.5(2)°; for the phenyl pendant at C(1), C-C is 1.526(3) Å; C-C-C(2,11a) are 109.70(15), 112.34(15)°.

**Table 3:** Crystal refinement data, *E*-**1**, **2**.

Compound	*E*-1	2
Formula	C_24_H_29_NO_4_	C_20_H_23_NO_3_
*F*_w_ (Da)	395.5	325.4
Crystal system	Orthorhombic	Monoclinic
Space group	*Pbca* (# 61)	*P*2_1_/*n* (#14 (variant))
*a* (Å)	12.321(1)	16.5518(6)
*b* (Å)	13.400(1)	6.1433(2)
*c* (Å)	25.698(3)	17.8034(6)
β (°)		106.931(4)
*V* (Å^3^)	4243(1)	1731.8(1)
T (K)	150	100
*Dc* (gcm^−3^)	1.238	1.24_8_
*Z* (f.u.)	8	4
μ (mm^−1^)	0.084	0.083
Specimen (mm^3^)	0.36 × 0.15 × 0.10	0.43 × 0.16 × 0.15
*T*_min/max_	0.77	0.97
2θ_max_ (deg.)	58	60
*N*_t_	50344	17112
*N*	5582 (0.030)	5052 (0.022)
*N*_o_ (*I* > 2σ(*I*))	3101	4391
*R*1 (*I* > 2σ(*I*))	0.053	0.043
*wR*2 (a(b))	0.15 (0.088)	0.111 (0.053, 0.58)
|Δρ_max_| (eÅ^−3^)	0.28	0.44

The conformations of the strings from C(5) to C(1), inclusive of the aromatic bond, are similar in both *E*-**1** and **2** (Figure 4a,b), but there are substantial differences between C(1)…C(5), with inversion at N(3) and a substantial difference in the angle sums about it: 343.3 in *E*-**1**, 323.6° in **2**, reflecting the difference in the N(3)-X(4) substituent and associated distances [1.450(2) in *E*-**1** (X = C), 1.4544(10) Å in **2** (X = O)]. It is not clear however, which of the two conformations seen in solution at low temperature for *E*-**1** and **2** correlate with the ring conformation for each seen in the solid state. Further studies are required to elucidate this. In each of these two compounds, as well as AYEKOF and SELXUC, with quaternary nitrogen atoms and with the double bond differently located or absent (Figure 4c,d), all of the macrocycle string lies to one side of the supporting aromatic ring plane. However, the conformations are different in each compound; a consequence of the diversity of string components and associated substituents. With related N,O-containing, nine-membered, reduced 2,6-benzoxazonine derivatives [28], the conformations adopted in the crystalline state (MOSXIB and MOSXOH [28]) were shown to be the same as those in solution.

High level calculations are required on the benzo-fused aza and oxaza ring systems in the present work in order to further probe the solution and solid state conformational landscape of these interesting heterocyclic systems.

## 3. Experimental Section

### 3.1. General

Microanalyses were carried out by the Central Science Laboratory, University of Tasmania, Hobart. Melting points were determined on a Yanagimoto Seisakusho micromelting point apparatus, and are uncorrected. Infrared spectra were recorded on a Digilab FTS-20E Fourier transform spectrometer. The ^1^H and ^13^C-NMR spectra were recorded on a Bruker AM-300 spectrometer at 300 and 75 MHz, respectively. Chemical shifts in ppm (δ) were measured relative to tetramethylsilane. Unless otherwise stated, the NMR spectra were measured in deuterated chloroform. Peaks are reported as singlet (s), broad singlet (bs), doublet (d), triplet (t), quartet (q) or multiplet (m). Where samples in the ^13^C-NMR exhibited several conformers, or isomers, the chemical shift for the major form is given first for each signal type; different forms are denoted with superscripts a,b or x,y in the ^l^H-NMR spectra. The mass spectra were recorded on either a VG MM 7070F or a Kratos Concept ISQ mass spectrometer operating at 70 eV. Peak intensities in parentheses are expressed as a percentage of the base peak. Analytical thin layer chromatography (t.l.c.) and preparative thin layer chromatography (p.t.l.c.) were performed on either Merck silica gel 60 F_254_ or Camag DSF-5 aluminium oxide. All column chromatography was performed under medium-pressure (“flash chromatography”) on either Merck silica gel 60, 230–400 mesh, or on type H aluminium oxide, 100–200 mesh. Preparative HPLC utilised a Dynamax-604 C18 reverse-phase column with a Waters 600 multisolvent delivery system and Waters 486 tunable UV detector. Samples were eluted at 10 mL/min and monitored at 254 nm. Mixtures of the chromatography solvents were made up by volume. Organic solvent extracts were dried with anhydrous sodium sulfate. Where reaction mixture solutions or solvent extracts were concentrated this refers to evaporation under reduced pressure on a rotary evaporator. When anhydrous conditions were necessary the glassware and solvents were dried and the additions or transfers were made via gas-tight syringes or stainless steel tubing, under a positive pressure of nitrogen. Light petroleum refers to a fraction boiling between 60–80 °C.

### 3.2. Synthesis of 6,7-Dimethoxy-2-methyl-1-(1-phenylethenyl)-1,2,3,4-tetrahydroisoquinoline (**3**)

A solution of 1-phenylethenylmagnesium bromide was prepared by the dropwise addition of 1‑bromostyrene (15.0 g, 81.9 mmol) to magnesium turnings (2.22 g, 91.3 mmol) in dry THF (200 mL) under nitrogen over 45 min. The mixture was heated briefly to initiate the reaction at the start of the addition with an iodine crystal present. The reaction temperature was then kept below 35 °C. The solution was stirred for 30 min. after the addition had ceased.

The solution of 1-phenylethenylmagnesium bromide in THF was cooled to <−50 °C in an acetone/liquid nitrogen bath and then the salt 6,7-dimethoxy-2-methyl-3,4-dihydroisoquinolinium iodide (13.52 g, 40.6 mmol; prepared from 6,7-dimethoxy-3,4-dihydroisoquinoline [23] on reaction with excess iodomethane in toluene at room temperature) was added. The mixture was stirred at <−50 °C for 90 min and then allowed to warm slowly to room temperature with stirring for 16 h before the careful addition of ice to decompose excess Grignard reagent. The mixture was basified by the addition of 40% aqueous potassium hydroxide and Et_2_O (150 mL) was added. The organic layer was decanted from the precipitated inorganic salts then centrifuged to remove any residual solids, washed with water (150 mL), and then concentrated. The residue was dissolved in Et_2_O (240 mL) which had been used, in three portions, to extract the inorganic solids and then the aqueous wash. The ether solution was washed with water (30 mL) then saturated brine (2 × 20 mL), and then dried and passed through a silica plug. Concentration of the solution afforded a solid which recrystallised to give the title compound **3** (11.11 g, 88%) as a pale yellow powder, mp 80–82 °C (EtOH). IR (KBr disc): 2768, 1514, 1256, 1217, 1143, 783 cm^−1^; ^l^H-NMR δ: 7.33–7.28 (m, 2ArH''), 7.22–7.17 (m, 3ArH''), 6.72 (s, ArH), 6.59 (s, ArH), 5.60 (d, *J =* 1.6 Hz, H2'), 5.34 (d, *J =* 1.6 Hz, H2'), 4.09 (s, H1), 3.84 (s, OCH_3_), 3.72 (s, OCH_3_), 3.10-3.02 (m, 2H), 2.66–2.45 (m, 2H), 2.33 (s, NCH_3_); ^13^C-NMR δ: 149.9, 147.9, 147.8, 140.3, 128.9, 128.5, 128.2, 128.0, 127.2, 119.1, 111.6, 110.6, 73.7, 56.3 (2 × OCH_3_), 52.0, 44.7, 29.4; *m*/*z*: 308 (M-H^+^, 0.5%; Calcd for C_20_H_22_NO_2_ 308.1650, found 308.1647), 206 (100), 190 (8), 162 (4), 132 (2), 103 (2), 77 (4); Anal. Calcd for C_20_H_23_NO_2_: C, 77.64; H, 7.49; N, 4.53. found: C, 77.67; H, 7.64; N, 4.42%.

### 3.3. Ethyl (E and Z)-9,10-Dimethoxy-3-methyl-6-phenyl-2,3,4,5-tetrahydro-1H-3-benzazonine-4-carboxylate (E**-1** and **Z-1**)

The base **3** (2.224 g, 7.19 mmol) was stirred in dry butanone (7 mL) under nitrogen with ethyl bromoacetate (1.81 g, 10.8 mmol) for 10 h at 50 °C. Concentration of the solution and trituration of the residue with Et_2_O (4 × 20 mL) afforded a gum which, on concentration from a solution in DCM, gave a mixture (*trans/cis* = 58:42) of the B-ring diastereomers of the 2-ethoxycarbonylmethylisoquino-linium bromide salts of **3** (1.183 g, 47%) as a tan hygroscopic powder; *trans* diastereomer (clear signals) ^13^C-NMR δ: 165.4, 143.4, 139.6, 131.4, 74.2–73.6 (bs), 63.2, 61.2, 56.5 (2 × OCH_3_), 53.8, 47.1, 23.8, 14.1; *cis* diastereomer (clear signals) ^13^C-NMR δ: 165.2, 142.5, 138.6, 130.7, 74.2–73.6 (bs), 63.0, 58.2, 56.5, 54.8, 49.8, 23.1, 14.1.

The *trans/cis* bromoacetate salt mixture above (307 mg, 0.644 mmol) was then stirred in dry acetonitrile (15 mL) under nitrogen at room temperature with DBU (170 mg, 0.84 mmol) for 5 h then the solution was concentrated. Purification of the residue by column chromatography on alumina with DCM/20% light petroleum afforded a pale yellow oil (206 mg, 81%). ^1^H-NMR analysis of this oil indicated a mixture of four components attributed to *Z-***1**/*E*-**1**:**6**/**8** in the ratio of 37:53:8:2. A portion (160 mg) of this mixture was purified by preparative reverse phase HPLC with acetonitrile/36% water to give:

(a): After 27.0 min, a mixture (9 mg) of two components (67:33) attributed to **6** and **8**. Compound **6** ^1^H-NMR δ: 7.42–7.25 (m, 5ArH''), 6.72 (s, ArH), 6.63 (s, ArH), 5.33 (s, H2'), 4.91 (s, H2'), 4.50 (d, *J =* 6.5 Hz, H2), 4.08-3.98 (m, 2H), 3.86 (s, OCH_3_), 3.82 (s, OCH_3_), 3.66 (d, *J =* 6.5 Hz, H1), 3.30–3.15 (m, 2H), 2.75–2.65 (m, 2H), 2.29 (s, NCH_3_), 1.18–1.12 (m, 3H); ^13^C-NMR (clear signals) δ: 172.6, 116.5, 67.0, 60.5, 53.3, 50.4, 46.6, 35.8, 15.1. Compound **8** ^l^H-NMR δ: 7.64 (dd, *J =* 1.4 Hz, 7.6 Hz, 2ArH), 7.42–7.25 (m, 3ArH), 7.06 (s, 1H), 6.96 (s, 1H), 6.89 (s, 1H), 6.80 (dd, *J =* 11.0, 17.0 Hz, 1H), 5.57 (dd, *J =* 1.0, 17.0 Hz, 1H), 5.17 (dd, *J =* 1.0, 11.0 Hz, 1H), 4.08–3.98 (m, 2H), 3.94 (s, OCH_3_), 3.91 (s, OCH_3_), 3.61 (s, 2H), 3.15 (s, 2H), 2.25 (s, NCH_3_), 1.20–1.14 (m, 3H); ^13^C-NMR (clear signals) δ: 171.6, 113.8, 60.8, 58.3, 55.2, 42.7, 14.8.

(b): After 32.8 min, the benzazonine isomer *Z*-**1** (50 mg) which recrystallised as colourless needles with mp 101–102 °C (EtOH). ^l^H-NMR (C_6_D_6_ at 75 °C) δ: 7.56 (d, *J =* 7.3 Hz, 2ArH''), 7.01–6.91 (m, 3ArH''), 6.74 (s, 1H), 6.60 (s, 1H), 6.56 (s, 1H), 4.10–3.82 (bs, CH_2_ of Et), 3.53 (s, OCH_3_), 3.45 (s, OCH_3_), 3.41–3.31 (m, H4), 3.01–2.94 (m, 4H), 2.66–2.57 (bs, H), 2.48 (s, NCH_3_), 2.42–2.36 (bs, H), 0.97–0.91 (m, 3H); ^13^C-NMR (C_6_D_6_) δ: 173.0, 150.0, 149.7, 141.3, 133.4, 132.6, 131.5, l17.6, 114.7, 66.0, 60.8, 58.0 (bs), 57.1, 57.0, 41.5, 37.5 (bs), 14.9, NCH_3_ not observed; ^l^H-NMR (CDCl_3_ at −50 °C): *conformer 1* δ: 6.88 (s, 1H), 6.54 (s, 1H), 6.38 (s, 1H), 3.98 (s, OCH_3_), 3.83 (s, OCH_3_), 2.36 (s, CH_3_), 1.41–1.35 (m, 3H); *conformer 2* δ: 6.82 (s, 1H), 6.67 (s, 1H), 6.45 (s, 1H), 3.93 (s, OCH_3_), 3.64 (s, OCH_3_), 2.69 (s, CH_3_), 0.87–0.81 (m, 3H); *m/z*: 395 (M^+^, 27%; Calcd. for C_24_H_29_NO_4_ 395.2096, found 395.2098), 377 (6), 322 (100), 291 (14), 265 (10); Anal. Calcd. For C_24_H_29_NO_4_: C, 72.88; H, 7.39; N, 3.54. Found: C, 72.69; H, 7.40; N, 3.36%.

(c): After 38.8 min, the benzazonine isomer *E*-**1** (54 mg) which recrystallised as colourless prisms with mp 124–125 °C (EtOH). ^l^H-NMR (CDCl_3_) δ: 7.52–7.49 (m, 2ArH''), 7.41–7.31 (m, 3ArH''), 6.78 (s, ArH), 6.61 (s, 1ArH and H7), 4.11–4.01 (m, 2H), 3.88 (s, OCH_3_), 3.83 (s, OCH_3_), 3.32 (dd, *J =* 3.3, 12.4 Hz, H4), 2.96–2.91 (m, 2H), 2.80–2.69 (m, 3H), 2.56–2.46 (m, 1H), 2.41 (s, NCH_3_), 1.24–1.18 (m, 3H); ^13^C-NMR δ: 174.3, 148.4, 147.4, 143.5, 141.7, 134.7, 130.3, 130.0, 129.1 (2C), 128.0, 127.4 (2C), l13.9, 112.4, 65.2, 60.7, 56.5 (2 × OCH_3_), 52.8, 45.1, 38.3, 34.8, 15.0; ^l^H-NMR (C_6_D_6_) δ: 7.56 (d, *J* = 8.4 Hz, 2ArH”), 7.30–7.17 (m, 3ArH”), 6.91 (s, H7), 6.65 (s, H8), 6.59 (s, H11), 3.97–3.87 (m, 2H), 3.56 (dd, *J* = 4.1, 12.0 Hz, H4), 3.49 (s, OCH_3_), 3.48 (s, OCH_3_), 3.35 (dd, *J =* 3.3, 15.5 Hz, H4), 3.07–2.88 (m, 4H), 2.78–2.70 (m, 1H), 2.47 (s, NCH_3_), 0.96–0.90 (m, 3H); ^13^C-NMR (C_6_D_6_) δ: 173.8, 149.6, 148.6, 143.5, 142.0, 134.7, 130.5, 113.9, 112.4, 65.5, 60.2, 55.9 (2 × OCH_3_), 53.2, 45.2, 38.8, 35.1, 14.7; *m/z*: 395 (M^+^, 29%; Calcd for C_24_H_29_NO_4_ 395.2096, found 395.2099), 377 (6), 322 (100), 291 (11), 265 (9); Anal. Calcd for C_24_H_29_NO_4_: C, 72.88; H, 7.39; N, 3.54. Found: C, 72.99; H, 7.58; N, 3.62%.

(ii): The bromoacetate salt above (297 mg, 0.623 mmol) was stirred in dry acetonitrile (15 mL) under nitrogen (10 mL) with DBU (0.12 g, 0.81 mmol) between −15 °C and −20 °C for 6 h. Workup as for (i) above afforded a mixture (200 mg, 81%) of *Z-***1**/*E*-**1**:**6** in the ratio of 38:54:8.

(iii): To the bromoacetate salt above (401 mg, 0.842 mmol) in refluxing dry acetonitrile (15 mL) was added DBU (170 mg, 1.1 mmol). The solution was refluxed for 1 h. Workup as for (i) above afforded a mixture (281 mg, 84%) of four components attributed to *Z*-**1**/*E*-**1**:**6**:**8** in the ratio of 36:35:11:18. Preparative reverse phase HPLC as for (i) afforded, after 27.0 min, a mixture (45 mg) of two components (37:63) attributed to **6** and **8**.

### 3.4. 6,7-Dimethoxy-2-methyl-1-(1-phenylethenyl)-1,2,3,4-tetrahydroisoquinoline N-oxide (**5**)

Whenever possible, solutions were cooled in ice throughout this procedure. To **3** (1.486 g, 4.803 mmol) in DCM (25 mL) at 0 °C was added a solution of *m-*CPBA (1.021 g, 5.936 mmol) in DCM (25 mL). The solution was stirred at 0 °C for 20 h then 5% aqueous sodium bicarbonate (30 mL) was added and the organic solvent was removed *in vacuo.* The cooled aqueous solution was washed with Et_2_O (3 × 10 mL) then made acidic to litmus with 3 M aqueous hydrochloric acid and washed with further Et_2_O (3 × 15 mL). The solution was basified with saturated aqueous sodium bicarbonate then saturated with sodium chloride and extracted with CHCl_3_ (3 × 25 mL). Drying and then concentration of the organic extracts afforded a 4:1 mixture of the *N*-oxide **5** and **2** (1.525 g). Purification of a portion (1.161 g) of this mixture by column chromatography on alumina with DCM/0-10% MeOH gave:

(a): A 78:22 mixture of **2** and **7** (156 mg, 14%).

(b): The *trans-*B-ring diastereomer of the tetrahydroisoquinoline *N*-oxide **5** (547 mg, 47%) as a cream hygroscopic solid. ^l^H-NMR **5**: 7.30 (s, 3ArH''), 7.07 (bs, *o-*ArH''), 6.71 (s, ArH), 6.62 (s, ArH), 5.61 (s, H1), 5.30 (s, 2H2'), 3.91 (s, OCH_3_), 3.83 (s, OCH_3_), 3.73–3.60 (m, 2H), 3.18–3.11 (m, 1H), 3.12 (s, NCH_3_), 2.65–2.58 (m, 1H); ^13^C-NMR δ: 149.3, 148.9, 147.8, 140.5, 129.4 (2C), 129.0 (2C), 128.0, 126.1, 124.8, 124.3, 111.6, 82.5, 59.8, 58.7, 56.7, 56.6, 24.9.

### 3.5. (Z)-9,10-Dimethoxy-3-methyl-6-phenyl-1,2,3,5-tetrahydro-4,3-benzoxazonine (**2**) and 7,8-Dimethoxy-3-methyl-1-(1-phenylethenyl)-1,3,4,5-tetrahydro-2,3-benzoxazepine (**7**)

To the tetrahydroisoquinoline **3** (2.056 g, 6.626 mmol) in DCM (40 mL) at 0 °C was added a solution of *m-*CPBA (1.40 g, 8.14 mmol) in DCM (60 mL). The solution was stirred at 0 °C for 40 h then 5% aqueous sodium bicarbonate (40 mL) was added and the organic solvent was removed *in vacuo.* The aqueous solution was washed with Et_2_O (3 × 25 mL) then made acidic to litmus with 3 M aqueous hydrochloric acid and washed with further Et_2_O (3 × 15 mL). The solution was basified with saturated aqueous sodium bicarbonate then saturated with sodium chloride and extracted with CHCl_3_ (3 × 25 mL). Drying and then concentration of the organic extracts, then column chromatography of the residue on alumina with DCM/0-10% MeOH, gave a 44:56 mixture of **2** and **7** (0.522 g, 24%) and the *trans* diastereomer of the *N*-oxide **5** (1.142 g, 53%). Preparative reverse phase HPLC of this mixture with acetonitrile/37% H_2_O gave:

(a): At 14.3 min, the 2,3-benzoxazepine **7** as colourless prisms with mp 96–97 °C (MeOH). ^l^H-NMR δ: 7.48–7.45 (m, *o-*ArH''*),* 7.33–7.25 (m, 3ArH''), 6.65 (s, ArH), 6.50 (s, ArH), 5.98 (s, H2'), 5.53 (s, H2'), 5.15 (s, H1), 3.85 (s, OCH_3_), 3.69 (s, OCH_3_), 3.45–3.37 (m, 1H), 3.12–3.04 (m, 1H), 2.91–2.86 (m, 1H), 2.72–2.67 (m, 1H), 2.66 (s, NCH_3_); ^13^C-NMR δ: 148.1, 147.9, 147.6, 140.6, 132.8, 130.4, 128.7 (2C), 128.0, 127.3 (2C), 117.3, 114.2, 111.7, 87.2, 60.6, 56.5, 56.3, 47.2, 33.7; *m/z*: 325 (M^+^, 12%; Calcd for C_20_H_23_NO_3_ 325.1677, found: 325.1670, 308(3), 266(100), 251(16), 237(10), 206(47); Anal. Calcd for C_20_H_23_NO_3_: C, 73.87; H, 7.13; N, 4.31. Found: C, 73.98; H, 7.29; N, 4.48%.

(b): At 17.1 min, the 4,3-benzoxazonine **2** as colourlesss prisms with mp 111–112 °C (MeOH). ^l^H-NMR δ: 7.55–7.52 (m, *o*-ArH'), 7.40–7.24 (3ArH'), 7.00 (s, H7), 6.75 (s, ArH), 6.63 (s, ArH), 4.51 (bs, H5), 3.89 (s, OCH_3_), 3.85 (s, OCH_3_), 2.94 (bs, 2H), 2.65 (s, NCH_3_), 2H of CH_2_CH_2_ not detected; ^13^C-NMR δ: 148.8, 147.5, 142.4, 142.1, 133.6, 131.3, 130.8, 129.1 (2C), 127.9, 126.8 (2C), 111.8, 111.1, 74.6, 63.0, 56.5, 56.4, 47.9, 32.9; *m/z*: 325 (M^+^, 13%; Calcd for C_20_H_23_NO_3_ 325.1677, found: 325.1670, 266 (93), 251 (18), 235 (7), 206 (100), 178 (12), 165 (17); Anal. Calcd for C_20_H_23_NO_3_: C, 73.87; H, 7.13; N, 4.31. Found: C, 73.84; H, 7.10; N, 4.44%. NMR analyses at −30 °C displayed two conformers in a 78:22 ratio. Assignable signals were: ^l^H-NMR δ: 7.11 and 6.92 (s, H7), 6.83 and 6.70 (s, ArH), 6.67 and 6.63 (s, ArH), 4.87 (d, *J* = 12.7 Hz, H5a), 4.47 (s, 2H5b), 4.32 (d, *J* = 12.7 Hz, H5a), 2.73 and 2.63 (s, NCH_3_); ^13^C-NMR δ: 132.4 and 131.7 (C7), 75.8 and 71.7 (C5), 63.0 and 62.9 (C2), 48.8 and 46.5 (NCH_3_), 31.7 and 34.3 (C1).

### 3.6. X-ray Structure Determinations

Full spheres of CCD area detector data were measured at “low”-temperature, (ω-scans, monochromatic Mo *Kα* radiation, λ = 0.7107_3_Å) yielding *N*_(__total)_ reflections, these being merged after “empirical”/multiscan absorption correction to *N* unique (*R*_int_ cited), *N*_o_ with *I* > 2σ(*I*) being considered “observed”. Full matrix least squares refinements on *F*^2^ were undertaken, anisotropic displacement parameter forms being refined for C,N,O, hydrogen atom treatment following a riding model, reflection weights being [σ^2^(*F*^2^) + (a*P*)^2^ + b*P*]^−^^1^ [*P* = (*F*_o_^2^ + 2*F*_c_^2^)/3]. Neutral atom complex scattering factors were employed, within the context of the SHELXL97 program [29]. Pertinent results are given in Table 3 and in Table 1 and Table 2 and Figures, non-hydrogen atom displacement ellipsoids, where shown, being at the 50% probability amplitude levels, hydrogen atoms, where shown, having arbitrary radii of 0.1 Å. Full* .*cif depositions, excluding structure factor amplitudes, are lodged with the Cambridge Crystallographic Data Centre, CCDC 972658 (**2**), 972659 (*E*-**1**). These data can be obtained free of charge via http://www.ccdc.cam.ac.uk/conts/retrieving.html (or from the CCDC, 12 Union Road, Cambridge CB2 1EZ, UK; Fax: +44 1223 336033; E-mail: deposit@ccdc.cam.ac.uk).

## 4. Conclusions

Derivatization on nitrogen in the common precursor tetrahydroisoquinoline base **3** and subsequent Stevens or Meisenheimer rearrangement under mild conditions allows access to the nine-membered 3‑benzazonine **1** (*E* and *Z* isomers, with the former predominant) as well as the analogous 4,3-benzoxazonine system **2** (*Z* only) with the same phenyl group disposition about the endocyclic double bond as for the *E*-benzazonine. The concerted [2,3] rearrangements involved are favoured when the reactive *N*-ylide or *N*-oxide functionality is *syn* to the 1-styrenyl substituent in the tetrahydro-isoquinoline precursor, while the competing [1,2] rearrangements to give the isomeric seven-membered ring compounds most probably proceed via a diradical intermediate. The nine-membered ring systems with the same endocyclic double bond geometry show some differences in ring conformations in the solid state from single crystal X-ray studies. Two conformations of *E*-**1** and **2** were detected by NMR studies in solution at low temperature. Application of catalytic enantioselective Meisenheimer [2,3] rearrangement methodology [30,31] to appropriately substituted 1-vinylic tetrahydroisoquinoline *N*-oxides is likely to be of further synthetic interest in the medium ring area.
